# A Validated Eco-Friendly High-Performance Liquid Chromatography Assay for Therapeutic Drug Monitoring of GS-441524 in Cats with Feline Infectious Peritonitis

**DOI:** 10.3390/pathogens15090913

**Published:** 2026-08-31

**Authors:** Stephen W. Cooke, Rachael Hammond, Danièlle A. Gunn-Moore

**Affiliations:** 1 The Microsampling Laboratory, 9 Plough Close, Shillingford, Wallingford OX10 7EX, UK; 2 The Royal (Dick) School of Veterinary Studies, The University of Edinburgh, Edinburgh EH25 9RG, UK; rachael.hammond@ed.ac.uk (R.H.); danielle.gunn-moore@ed.ac.uk (D.A.G.-M.)

**Keywords:** GS-441524, remdesivir, feline infectious peritonitis, treatment, therapeutic drug monitoring, dosage, plasma concentration, cat, HPLC

## Abstract

Feline infectious peritonitis (FIP) is responsive to treatment with the adenosine analogue GS-441524 (GS-44) and its prodrug, remdesivir (REM); both are now available on veterinary prescription in many countries. Therapeutic drug monitoring (TDM) of GS-44 has the potential to support dose selection for individual cats; however, TDM assays are currently only offered by one UK-based laboratory. This study describes a simple, cost-effective, and environmentally conscious high-performance liquid chromatography (HPLC) method for the quantification of GS-44 in feline plasma or serum. The method was validated in accordance with the International Council for Harmonisation M10 guidelines for bioanalytical methods. Calibration standards demonstrated linearity across a range of 2.59 to 5048.28 ng/mL, R^2^ = 0.9880, with a lower limit of quantification of 2.59 ng/mL and upper limit of quantification of 5048 ng/mL; this is equivalent to an assay range of 0.06 to 208 µM. Precision, accuracy and spike recovery were within ±15% for 14 of 17 standard concentrations (±20% at the lowest three). Carry-over, dilution integrity, and analyte stability under common storage conditions all met the method’s requirements. This is a simple, robust and accurate eco-friendly method suitable for adoption by diagnostic laboratories, enabling routine TDM for cats undergoing treatment for FIP with GS-44 and/or REM.

## 1. Introduction

Remdesivir (REM) and GS-441524 (GS-44) are anti-viral agents that are safe and effective when used to treat cats suffering from the various forms of feline infectious peritonitis (FIP) [1,2]. Both drugs are now legally available for prescription by veterinary surgeons in many countries from licensed veterinary pharmaceutical compounding companies.

The treatment regimens being used for these drugs are based on data derived from studies involving in vitro cell cultures and healthy cats [3,4,5,6,7,8] as well as retrospective reports of results from cats treated with legally sourced GS-44 and/or REM [4,7,8,9,10,11,12,13,14,15,16,17,18,19,20] or unverifiable compounds sourced illegally [21,22,23,24,25]. To date, published pharmacokinetic (PK) data for these drugs being administered under veterinary supervision to cats with clinical FIP are sparse (*n* = 6 [PK data] *n* = 22 [single sample data]) [8] and there is a need for robust investigation into dosages appropriate for each clinical presentation of FIP [8]. Given the limited predictive PK/pharmacodynamic (PD) data available for clinical cases, accurate quantification of GS-44 concentrations is an essential alternative to inform evidence-based dosing and therapeutic monitoring.

Therapeutic Drug Monitoring (TDM) is used to monitor the plasma concentration of target drugs to optimise treatment [26]; this is primarily to prevent under- or over-dosage, but also to support investigations into causes of failure in response to medication, side effects or relapses from remission.

TDM of GS-44 requires a laboratory method readily available to clinicians which can report results both rapidly (<7 days) and cost-effectively. At present, high performance liquid chromatography combined with mass spectroscopy (HPLC-MS or HPLC-MS/MS [27,28,29,30]) is the method most used by researchers; however, this utilises expensive and complicated equipment, that needs to be run by specialist technicians, it uses significant amounts of environmentally sensitive resources and is not (June 2026) offered for clinical use.

The aim of this paper is to describe the validation of a simple, low cost, effective HPLC method without using MS and using sparing amounts of environmentally sensitive resources, which may find a place in commercial diagnostic laboratories and provide clinicians with access to this important resource.

We also report the GS-44 assay results of 1034 samples originating from 361 cats undergoing treatment for the various clinical presentations of FIP.

## 2. Materials and Methods

### 2.1. Sample Origin and Handling

Feline serum (spontaneous clotting or serum gel) and plasma samples (ethylenediaminetetraacetic acid [EDTA] and/or heparin anticoagulated) were obtained as residual diagnostic material from routine clinical submissions to the Easter Bush Pathology Laboratory, Royal (Dick) School of Veterinary Studies, University of Edinburgh, and as diagnostic samples originating from cats receiving legally prescribed REM or GS-44 for the management of FIP. The use of residual clinical samples for assay development and validation was approved by the Veterinary Ethical Review Committee (VERC) of the University of Edinburgh, UK (VERC 63.22; approved 4 December 2022). Following diagnostic testing, residual material was transferred to The Microsampling Laboratory for method development and validation.

### 2.2. Chemicals, Reagents and Expendables

All solvents (including ultra-pure water) and chemicals were of HPLC grade (filtered at 0.22 µm) and purchased from LaserChrom HPLC Laboratories, London, UK, except for GS-44 (CAS: 1191237-69-0, purity > 99%, stored at −20 °C in its original shipping container), which was purchased from MedChemTronica AB, Stockholm, Sweden.

### 2.3. Preparation of Stock and Standard Solutions

Methanol (100%) was used to prepare a 36,600 ng/mL stock solution of GS-44, which was stored at −20 °C. Seventeen standard solutions, ranging from 5048.28 to 2.59 ng/mL GS-44, were prepared from the stock solution diluted with the HPLC mobile phase (MP) described below. Methanol was not used as a diluent for the standards as, during method development, excessive perturbation of the chromatogram’s baseline and peak broadening/shouldering occurred when using methanol, artefacts not exhibited when using MP as the diluent; this appeared to be a column specific phenomenon. Standard solutions were prepared in 2 mL HPLC sample vials and stored between 0 °C and +6 °C (laboratory refrigerator) and briefly vortexed before each assay sequence. Standard solutions were assayed together with study samples and discarded if their calculated concentration deviated more than 15% (more than 20% for the three lowest standard concentrations) from the expected values. Fresh standard sets were prepared as necessary (approximately every 6 months).

### 2.4. Extraction and Sample Preparation

For this paper, the term “sample” refers to plasma from EDTA- or heparin-anticoagulated blood or serum.

The GS-44 blood-plasma partitioning ratio for cats is believed to be 1.2 (five other species returned similar values [31]); thus, for completely haemolysed samples, assay values were likely to be approximately 20% higher than those obtained if haemolysis was not present. For this study, if red blood cells could not be separated from haemolysed samples by centrifugation, i.e., complete haemolysis had occurred, these samples were excluded.

Molecules causing icterus and lipaemia in samples would not cause interference with the method [32,33], so these artefacts were not considered to be interfering factors.

Each sample was prepared at 2 dilutions by mixing 220 µL methanol (to remove proteins by coagulation) with (a) 20 µL of sample (1 in 12 dilution) and (b) 40 µL of sample (1 in 6.5 dilution) using a positive pressure pipettor (Gilson Microman; Gilson UK, Dunstable. UK) to prevent viscous samples causing measuring errors. After vortexing twice for 10 s with a 30 s interval and centrifugation at a relative centrifugal force of 7700 g for 6 min, 210 µL of the resulting supernatant was transferred to a fresh 1.5 mL vial and evaporated to dryness under vacuum (GeneVac EZ-2, circa 60 min at ≤40 °C). The residue was redissolved in 210 µL MP, using an ultrasonic bath (5 min at ≤40 °C) and vortexing, and then filtered through a 4 mm 0.22 µm nylon syringe filter into a 0.3 mL HPLC vial for analysis.

### 2.5. Instrumentation and Chromatographic Condition

A Dionex (Fisher Scientific UK Ltd., Loughborough, UK) UltiMate RS 3000 UHPLC system, consisting of a four-channel MP vacuum degasser, temperature-controlled auto-sampler (20 °C), twin pumps, column oven (column 40 °C; post-column 23 °C) and a four-channel UV diode array detector (DAD) was used. Instrument control, data collection and chromatogram analysis were performed by Chromeleon v 7.2 (Fisher Scientific UK Ltd., Loughborough, UK) software. The sample injection volume was 40 µL, with needle washing using MP between injections.

The GS-44 molecule is highly polar (XlogP3-AA = −1.4 [34]) and so requires a high aqueous content MP to increase its retention time on C18 solid-phase columns sufficiently to ensure adequate peak separation from other sample components. The Waters XSelect HSS T3 100Å, 2.5 µm, 4.6 mm × 150 mm column (Waters Ltd., Wilmslow, UK), was selected as its solid phase was designed to be used with an MP containing up to 100% water and, during method development, provided satisfactory separation of the GS-44 peak from other sample components which also absorbed UV wavelengths between 220 and 260 nm. Elution was isocratic with a 1.5 mL/min MP flowrate and run time of 13 min, and pressure ± <5 bar (nominal working pressure circa 360 bar).

Sequences (a series of assay runs performed on a single day) were made up of repeats of assay runs “standard a, sample 1, sample 2, sample 3, standard b”, etc. This ensured that any carry-over from a sample was visually identifiable (the presence of “ghost peaks”) and that the column was flushed on a regular basis to maintain its equilibrium (exhibited by baseline stability and/or no increase in baseline noise (BLN)).

The MP was made up freshly for each sequence and consisted of a water: acetonitrile (ACN): formic acid (FA) mixture, nominally 96:4:0.1 *v*/*v*, made by mixing, in a 500 mL volumetric flask, circa 300 mL water with 20 mL ACN and 500 µL 98% FA, and then increasing the volume up to 500 mL with water. Ambient temperature was 21 ± 5 °C.

Detection of GS-44 was achieved by identifying a discrete peak eluting at 4.6 ± 0.3 min, which was quantified by measuring light absorption at 240 ± 5 nm (wide slit, 5 Hz sampling rate); the area under the peak (AUP) in absorption units (mAU) was determined by the software. Two standards run at the start of a sequence confirmed the elution time for the GS-44 peak.

The standard GS-44 concentrations (ng/mL) were calculated from the AUP using the external standard linear regression formula of best fit [4] and then converted to µM (ng/mL/291.26 [molecular weight of GS-44]) and sample concentration (µM × sample dilution factor).

### 2.6. Method Validation

The method was validated using study samples (*n* = 1034), blank samples (plasma or serum from cats known as not receiving GS-44 or REM; *n* = 15) and standards (*n* = 17); it was assessed for selectivity, specificity, sensitivity, LLOQ, calibration curve (and linearity), accuracy, precision, matrix effect, carry-over and stability in accordance with the biological method validation guidelines prepared by the International Council for Harmonisation of Technical Requirements for Pharmaceuticals for Human Use, Bioanalytical method full validation and study sample analysis, M10 adopted 24 May 2022 (ICH M10) [35].

Due to only small volumes of blank cat plasma being available for use as the matrix for the calibration and QC samples, it was not possible to use this matrix for standard and QC samples during the entirety of the study; instead, after validation, the standard solutions were substituted. This decision was supported by the equivalent performance of the standard solutions as used during method validation when compared with the blank plasma standard results.

Use of an external standard was selected as there was no likely benefit expected from using an internal standard with this uncomplicated sample preparation method [4,36].

### 2.7. Selectivity, Specificity and Sample Processing

Selectivity and specificity were assessed using both blank and study cat samples with and without being spiked with GS-44. The retention time of GS-44 with blank cat samples spiked with GS-44 was compared with that obtained for standards and study samples.

**Figure 1:**
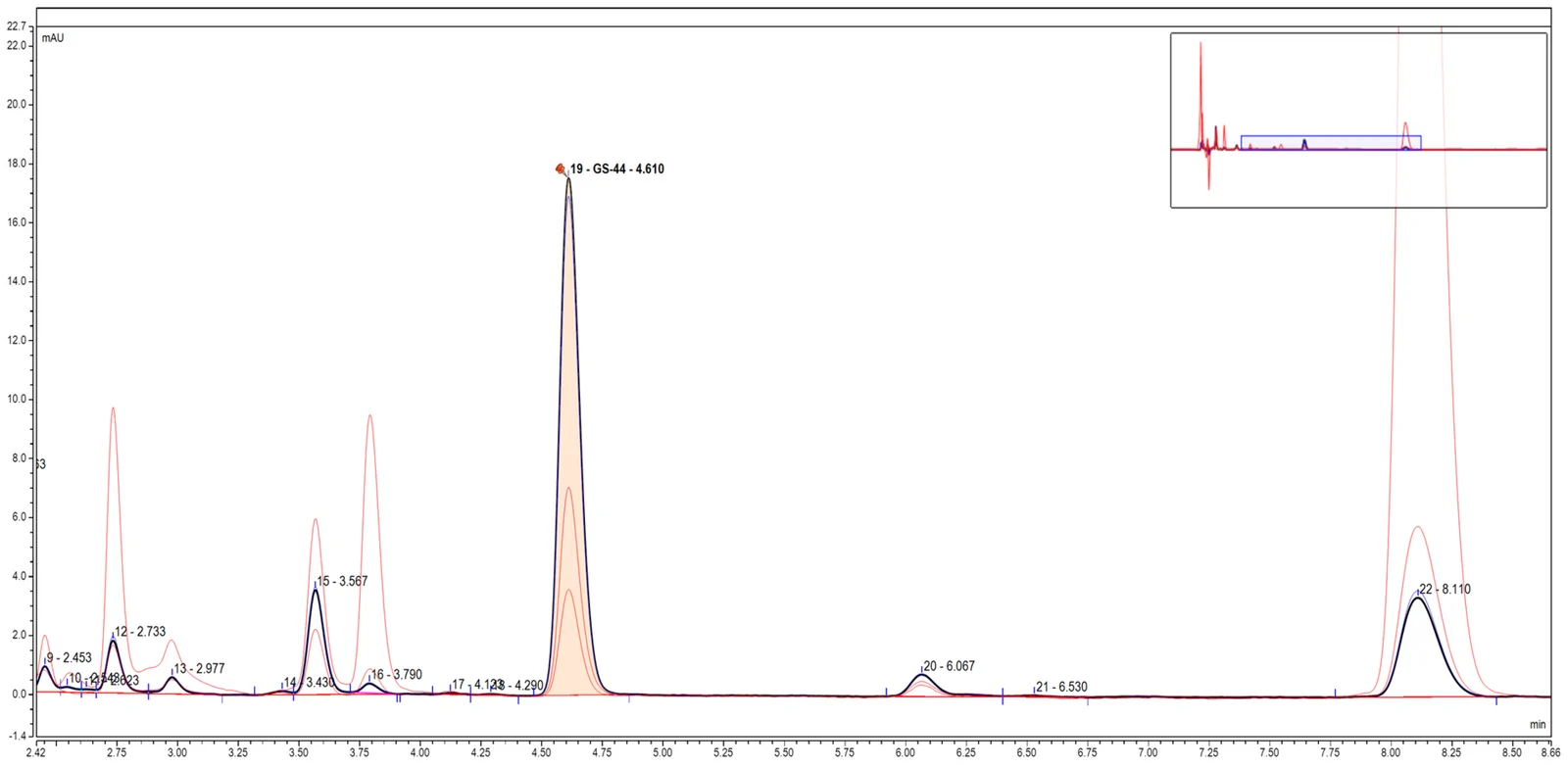
Chromatogram of a study sample, showing all four absorption channels recorded during the assay. Absorption wavelengths were set at 240 ± 5 nm (black), 240 ± 10 nm (blue), 230 ± 10 nm (red), and 250 ± 10 nm (red). The elution profiles and relative absorbance ratios across these wavelengths and bandwidths were visually compared at the upslope, apex, and downslope of the GS-441524 (GS-44) peak at 4.61 m (highlighted in tan) to confirm spectral homogeneity and rule out the presence of interfering co-elutions (such differences are seen for peaks eluting at different times and which were not GS-44). The area under the peak (AUP) of channel one (black) was used to quantify the GS-44 concentration in the sample. A complete chromatogram is depicted in the window to the upper right; the blue box represents limits of the magnified portion.

Method development determined the HPLC conditions required to ensure that the GS-44 peak occurred at a point in the sample chromatogram where peaks resulting from other components present in the study samples would not coincide. Naturally occurring substances as well as the gamut of possible exogenous compounds (including veterinary medications and owner-administered compounds) were too numerous to be individually tested, so the method stipulated real-time examination of each chromatogram by one author (SWC) to confirm that the GS-44 peak was unadulterated. Peak purity and the absence of co-eluting impurities were evaluated using simultaneous multi-channel data collection. Absorption was recorded at 240 ± 5 nm, 240 ± 10 nm, 230 ± 10 nm, and 250 ± 10 nm. The elution profiles and relative absorbance ratios across these wavelengths and bandwidths were visually compared at the upslope, apex, and downslope of the GS-44 peak to confirm spectral homogeneity and rule out the presence of interfering co-elutions (Figure 1). At this point, there would be no interfering peaks (satisfying the requirement for interfering peaks being <20% of the LLOQ response).

For each sample, the two dilutions were assayed within the same sequence separated by at least three other assay runs. If the calculated GS-44 concentration values differed by more than 15%, then either one or both dilutions were re-run. If the repeats could not correct the variation (by identifying the outlier value), then a fresh dilution pair was made up for that sample and assayed.

The two dilutions acted as a self-check for any errors in sample preparation or the assay process (including injection reproducibility). If the results of the two dilutions fell within 15% of each other, the sample assay results were reported as the average of the two results ±1 one standard deviation (SD).

Extraction recovery was assessed using spike recovery experiments. Both normal samples (*n* = 11, known not to contain GS-44) and study samples (*n* = 5, pre-assayed and containing known amounts of GS-44) were spiked with solutions of GS-44 prepared to produce the final concentrations of 2.86, 5.71 and 11.42 µM when 4 µL of each was added to a 40 µL sample. Extraction recovery efficiency was calculated as the percentage difference between the observed and expected concentrations.

### 2.8. Calibration Curve, Range of Values, Upper Limit of Detection (ULOD) and Lower Limit of Quantification (LLOQ)

Seventeen standard solutions were prepared with a range of 5043.39 to 2.59 ng/mL.

The calibration curve was determined by assaying the standard solutions and plotting the AUP in mAU against the expected GS-44 concentration in ng/mL. Each of the 17 standards was assayed at least four times (range 4–21). A calibration curve and correlation coefficient were determined as a simple linear regression model of these results. Similarly, a cumulative SD was calculated for each standard and plotted against the calculated concentration and the resulting simple linear regression model was used to calculate an appropriate SD for the sample assay results [37].

For the range of values, the ULOQ was set at 5045 ng/mL, equivalent to a plasma concentration of 208 µM at a 1 in 12 dilution, which is a value greater than sample values from clinical cases (with a maximum assay value of 49.2 µM in our series) and greater than the GS-44 concentration reported to not cause cytotoxic effects in vitro (100 µM [3,6]); this is a value not anticipated to be achieved in clinical cases, but is not impossible.

The LLOQ was calculated using the BLN height (mAU) as determined by the software, over multiple runs of the three lowest standard concentrations. The LLOQ was defined as the standard concentration with a GS-44 peak height of >five times the maximum BLN value.

### 2.9. Precision and Accuracy

Precision and accuracy were evaluated by analysing at least four different standards within each day’s sequence (intra-day) and the same standards over different day’s sequences (inter-day). Accuracy was expressed as the percentage deviation of the mean measured concentration from the nominal concentration and precision as the coefficient of variation (CV% = [standard deviation/mean] × 100). Acceptable criteria were accuracy within 85–115% (80–120% for the lowest three standard concentrations) and precision (CV) ≤15% (≤20% for the lowest three standards).

### 2.10. Carry-Over

Carry-over occurs where either (i) a sample component had a retention time greater than the run time, thus producing a peak in the subsequent run, or (ii) where GS-44 was not completely eluted from the solid phase during a run, so it was released in the subsequent run and appeared as an unidentified peak. These were assessed by observing sequential runs, using either study samples or the highest concentration standard GS-44, followed by MP.

### 2.11. Dilution Integrity

Standard solution dilution integrity was assessed by comparing standard solution assay results with expected values for different batches of standard solutions.

### 2.12. Stability

The stability of the standard solutions was assessed by monitoring the standard results on each day. If the AUP of any standard differed by >15% (>20% for the three lowest standards) of that recorded for the freshly prepared standard, that standard was discarded. If >25% of the standards from that set were excluded for this reason, then the remainder were discarded and a new set prepared.

Stock solution stability was monitored by comparing the AUPs of each new set of standards with the initial results for each previous standard set.

Sample stability was assessed by repeating sample assays after samples had been held at room temperature (RT) for 4 days, RT 4 days then fridge temperature for 4 days or RT 4 days, fridge temperature 4 days then frozen for 4 days.

### 2.13. Statistical Analysis

Statistical analysis was carried out using Excel (Microsoft Corporation, Redmond, WA, USA) and GraphPad Prism v10.6.1 (892) (GraphPad Software, Boston, MA, USA); *p*-values <0.05 were considered statistically significant.

## 3. Results

### 3.1. Validations

#### Selectivity and Specificity

Chromatograms of sample, standard GS-44 solutions and GS-44 spiked blank cat samples produced GS-44 peaks at an elution time of 4.6, which varied slightly (±0.3 min) between days, depending on the temperature at which the MP was prepared (volumetric effect of, mainly, ACN).

For blank cat samples, the baseline was near to or at 0.0 mAU, stable for the baseline time between 2.5 and 9 min, exhibited a BLN of <0.025 mAU and had no peaks evident at the expected GS-44 elution time 4.6 ± 0.3 min. For spiked samples, the GS-44 AUP corresponded to <±20% of the expected value and no endogenous peaks were seen to interfere with the GS-44 peak.

**Figure 2:**
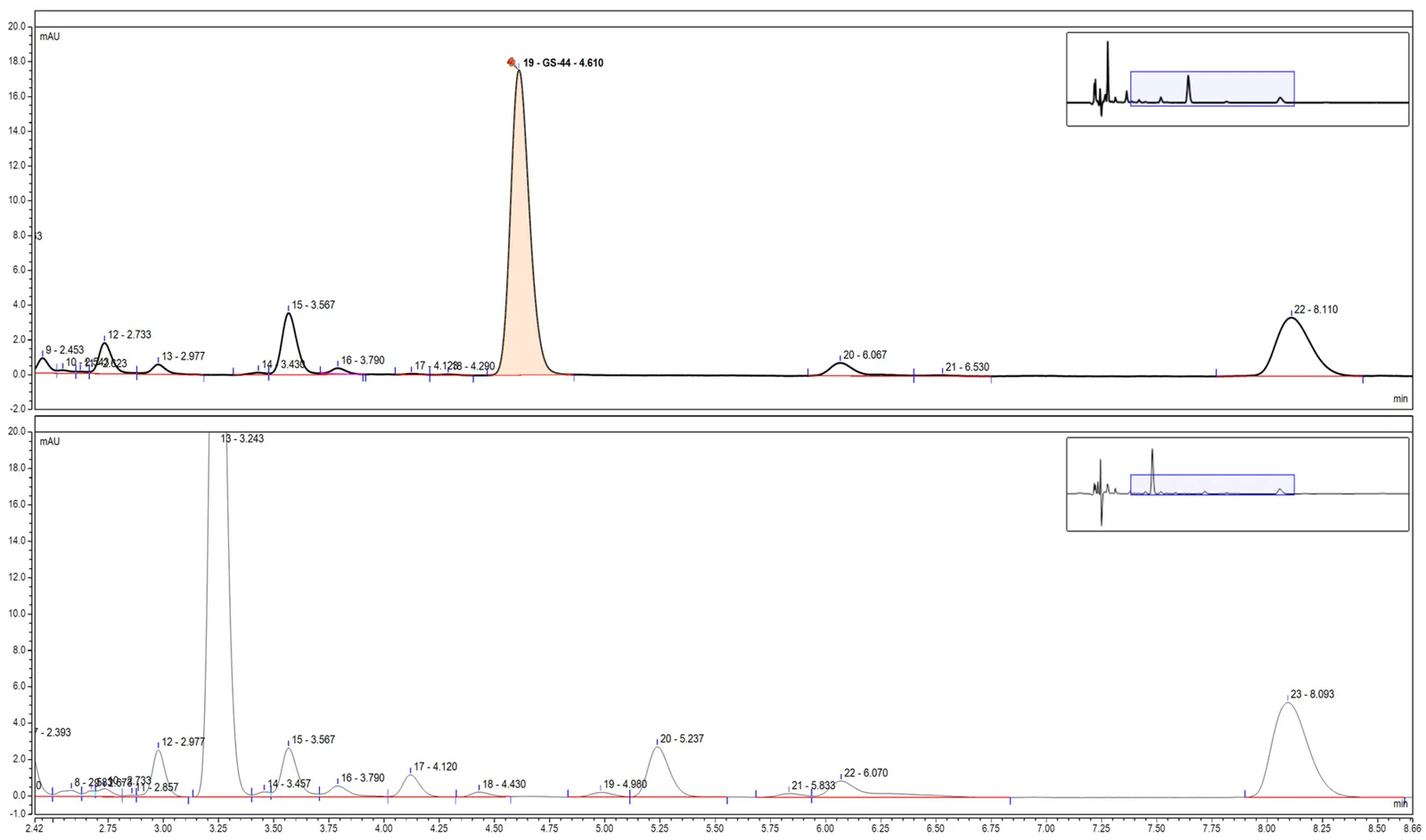
Chromatogram of (**top**) a study cat sample from a cat known to have received GS-441524 (GS-44) and (**bottom**) a blank cat plasma from a cat known not to have received GS-44. GS-44, peak 19, is shown coloured tan. Chromatograms are the black and grey lines, baseline is shown red. A complete chromatogram is depicted in the window to the upper right of each; the blue box represents limits of magnified portion. Peak numbering: sequential peak number (-) elution time in minutes. Absorption measured at 240 ± 5 nm.

Representative examples of chromatograms of blank cat plasma and a study sample are shown in Figure 2. For the blank cat plasma there are no peaks that occupy the same elution time as the GS-44 in the study sample. Spiking GS-44 into a sample containing endogenous GS-44 did not change the elution time, and did not produce peak fronting, tailing, splitting, shouldering, base broadening or retention time variation. Similar results were observed in the spike recovery experiments performed on another 11 study samples. This shows that the assay is selective and specific.

### 3.2. Matrix Effect

The GS-44 peaks were not overtly affected by other compounds present in the samples, as demonstrated by the absence of changes in peak characteristics and elution times across the study sample chromatograms at the four-channel wavelength values and bandwidth, by visual inspection of each chromatogram.

### 3.3. Calibration Curve, Range of Values, Upper Limit of Detection (ULOD) and Lower Limit of Quantification (LLOQ)

The calibration data (Table 1) best fitted to a simple linear regression model, y = 0.00301x, with the intercept set to 0.0, r^2^ = 0.9974. A second-order model was also generated; however, this failed to correctly fit the two lowest standard concentration results and so was discarded.

The SD data (Table 1) best fitted y = 0.0521x − 0.0071 and r^2^ = 0.9880.

Values for BLN never exceeded 0.026 mAU; this value was used to calculate LLOQ.

The spike recovery study produced an average recovery of 95%, ranging from 86 to 110%; the absolute bias was 5%. The recovery calculated from the precision experiment was 99% (range 89–105%) with an estimated absolute bias of 1%.

From the standard assays, the LLOQ was set at 2.59 ng/mL (Table 2), equivalent to a plasma concentration of 0.06 µM at a 1 in 6.5 dilution. Table 2 shows the justification for this choice with calculations for assays (*n* = 19) of the lower three standard dilutions and their respective absorption, peak height and BLN values.

### 3.4. Precision and Accuracy

Trueness (as bias) was <5% for all except the lower two dilutions, which were <10%, and precision (as coefficient of variation [CV]) was <5% except for the lower two dilutions, which were <15%.

### 3.5. Carry-Over

Examination of the chromatograms obtained using blank plasma or an MP run immediately after both standard and sample assay runs did not show any peaks at 4.6 ± 0.3 min, indicating that neither GS-44 nor other sample components were retained by the column for more than 18 m; therefore, carry-over did not occur with this run time and GS-44 dilution range.

### 3.6. Stability

Stock solution stability was acceptable, with a reduction in stock GS-44 content <5% over a 16-month period.

Standard solutions were also very stable; the average time between batches being replaced with fresh ones was 43 days. The commonest reason for standard sets being discarded was because their volume was depleted from use. During that time, a slight increase in concentration was noted (typically 1–3%) and was believed to be due to evaporation during use.

Sample stability was also acceptable, with variation between stored and fresh assay sample results <11% over a 10-day period at temperatures ranging from −20 °C (freezer), 0–6 °C (fridge), 20 °C (autosampler) and ambient.

### 3.7. Clinical Application

**Figure 3:**
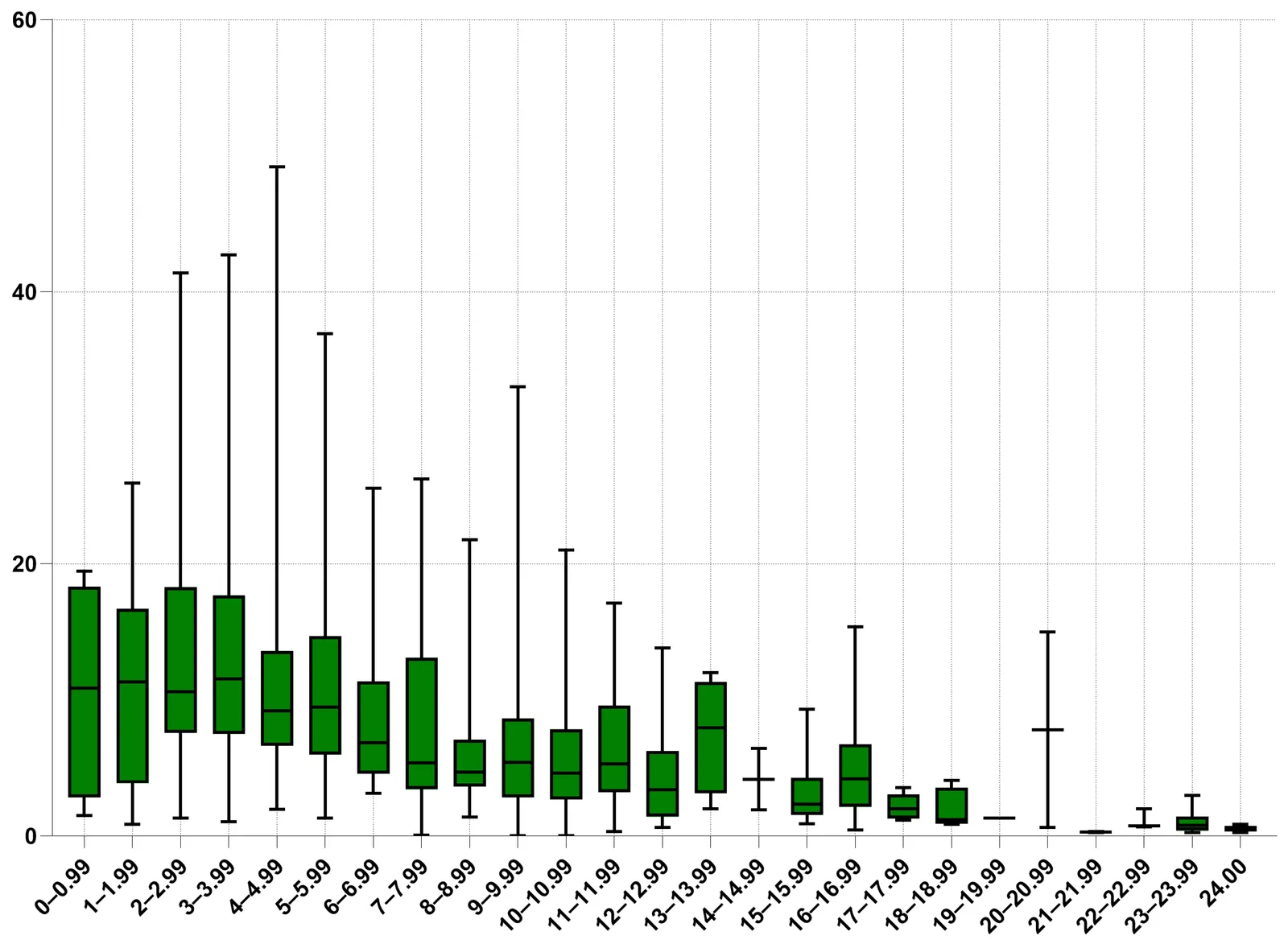
For 1034 study samples originating from 361 individual cats: y-axis—sample GS-441524 concentration (µM) for (x-axis) sample time (hours) after drug administration are shown aggregated into one-hour time bins. Bar and whisker plot: whiskers show minimum to maximum values; the green bar encompasses the 25th to 75th percentile; the black bar in the green area is the median value.

A total of 1034 samples originating from 361 individual cats were assayed. The results of sample GS-44 concentration (µM) were assigned into 24 one-hour bins, representing the sample time after dose administration for sequential one-hour periods (e.g., bin 1 = 0–59 min, bin 2 = 60–119 min etc.); these were plotted as a bar and whisker plot (Figure 3). The black bar indicates the range of values, the green bar indicates the 25% to 75% percentiles with a solid intermediate bar being the median value.

Of note is the fact that a wide variation in sample GS-44 concentration was observed, and this is reported and discussed elsewhere [38].

## 4. Discussion

This study successfully developed and validated an HPLC method using ultraviolet detection for the quantification of GS-44 in blood samples from cats being treated with the compound for FIP, and satisfied the standards set out by the International Council for Harmonisation in the document M10, Technical Requirements for Pharmaceuticals for Human Use, Bioanalytical method full validation and study sample analysis [35].

Both endogenous (sample) GS-44 and spike GS-44 were eluted from spiked samples already containing GS-44 with the same retention time, indicating that the assay is both selective and specific.

Because an unidentified compound (unrelated to GS-44), found in both study and blank cat samples, produced a peak eluting at 11 min, a run time of 13 min was chosen to prevent this compound from contaminating subsequent chromatograms.

Due to the complexity of FIP’s clinical presentation and systemic effects it would be expected for some of the study samples to contain exogenous compounds such as corticosteroids, antibiotics, appetite stimulants, analgesics, sedatives or other antiviral compounds [4]; however, it would be singularly coincidental if any such compounds both eluted with exactly the same retention time as GS-44 (as found with this method) and absorbed light between 230 and 250 nm. Observing each chromatogram as it developed allowed the operator to ensure that no compounds with elution times ± 0.3 min of the GS-44 value produced overlapping peaks and subsequent GS-44 peak artefacts. None were observed.

There was very good separation of GS-44 from other components of cat plasma noted for all the study samples and the method produced consistently reproducible results using a simple sample preparation technique and straight-forward isocratic MP elution.

The HPLC method described here also satisfies the requirements of a, comparatively, eco-friendly method [39]; ACN is a toxic substance (also designated methyl cyanide) that requires either professional recycling using fractional distillation (an expensive and energy intensive method) or (as used in this study) chemical neutralisation using sodium hydroxide [40], so it was desirable to have the smallest possible concentration of ACN in the MP. The study method uses 0.78 mL ACN per run, whilst one recent non-MS HPLC method [4] used 5.39 mL ACN per run and another, using MS [41], used 2.61 mL per run. Assays utilising HPLC-MS or HPLC-MS/MS require equipment that has to be maintained at vacuum permanently (using electrical energy and producing waste heat and noise 24/7), uses high purity nitrogen, argon or helium (which are energy demanding to manufacture), requires the use of high purity solvents and specialized sample vials (more energy demanding than LC equivalents) and may also require radioactive internal standards (energy demanding to produce). Developing a method using stand-alone HPLC obviates the need to use equipment dependent on such environmentally costly complexities.

It is anticipated that method development will continue with the use of UHPLC-specific columns to reduce run times and reagent usage, thus further reducing the environmental impact of the assay.

Other nucleoside analogue antiviral drugs such as molnupiravir (EIDD-2801) have been used to treat FIP [5] and the method described in this paper is currently being modified and used to support TDM for this compound by assaying its parent drug, N4-hydroxycytidine (NHC, EIDD-1931), which is also used therapeutically.

The requirement of observing the development of each chromatograph restricts the commercial use slightly, as the method is not of a “walk-away” nature, so trained operators would be required to observe throughout the sample sequence each day.

The clinical application of the method to generate TDM data, appears to be justified as the sample results collected for this paper (the study continues as of June 2026) show a wide range of values for each one-hour time bin after drug administration (Figure 3). This wide variation suggests a similarly wide range of metabolic processes being carried out in cats affected by FIP and the consequent variability with which GS-44 is absorbed and/or eliminated after administration. This is not unexpected as FIP has at least four phenotypes (including cats expressing more than one) and affects a disparate population of age, sex, breed, husbandry and immunological competence. Such variations may be translated into how efficiently GS-44 is presented to its intended site of action (monocytes and macrophages) and the inherent potential for sub-optimal antiviral effects leading to failed treatments or relapse from remission. As a clinical tool, TDM is well-suited to investigate and quantify these variations.

## 5. Conclusions

The study describes an assay method for plasma and serum GS-44 which satisfies the practical requirements of both diagnostic and research laboratories in respect of cost effectiveness, usage of readily accessible technology, simple and eco-friendly method and ease of implementation. We hope that this will encourage, depending on the method being adopted by clinical laboratories, a more widespread use of TDM for monitoring the tissue concentrations of GS-44 (and perhaps similar drugs) during the treatment of FIP. This, in turn, will make it possible to populate sufficiently large data sets representing all the variables inherent in the clinical expression of FIP and construct meaningful population PK models, further refining the treatment of this disease.

## Figures and Tables

**Table 1:** Standard GS-441524 calibration dilutions and assay results, including percentage residual standard deviation (RSD%) and standard deviation (SD) in both ng/mL and µM.

Standard Set Used	Number of Standard Assays	Expected Standard Concentration ng/mL	SD for Each Standard ng/mL	Mean for Each Standard ng/mL	RSD% for Each Standard	Expected Concentration µM	SD for Each Standard µM
1	13	5048.28	259.68	5043.39	5.15	17.333	0.892
2	21	3527.71	195.78	3504.24	5.59	12.112	0.672
3	18	1853.16	78.09	1946.70	4.01	6.363	0.268
4	11	950.65	38.17	950.90	4.01	3.264	0.131
5	11	834.53	36.65	828.14	4.43	2.865	0.126
6	21	717.65	38.92	712.33	5.46	2.464	0.134
7	18	600.00	11.28	619.47	1.82	2.060	0.039
8	17	481.58	26.73	466.95	5.72	1.653	0.092
9	17	362.38	23.97	366.64	6.54	1.244	0.082
10	12	242.38	14.52	240.93	6.03	0.832	0.050
11	13	121.19	7.66	126.33	6.06	0.416	0.026
12	19	60.60	3.79	59.61	6.36	0.208	0.013
13	2	32.11	1.01	30.85	3.27	0.110	0.003
14	14	16.00	1.53	15.74	9.75	0.055	0.005
15	4	10.36	0.33	9.78	3.41	0.036	0.001
16	4	5.18	0.17	4.97	3.39	0.018	0.001
17	10	2.59	0.23	2.40	9.52	0.009	0.001

**Table 2:** Lowest three GS-441524 standard assay results and LLOQ selection criteria. LLOQ 2.59 ng/mL, peak height >5 × baseline noise (BLN) (Ratio height to BLN).

Standard	GS-44 Conc, Expected; ng/mL	GS-44 Assay Calculated; ng/mL	As% of Expected	AUP; mAU/min	Height; mAU	BLN Height; mAU	Ratio Height to BLN
15	10.36	9.60	93%	0.0289	0.340	0.021	16.190
15	10.36	9.97	96%	0.0300	0.340	0.016	21.250
15	10.36	7.41	72%	0.0223	0.260	0.011	23.636
15	10.36	9.40	91%	0.0283	0.330	0.012	27.500
16	5.18	5.05	98%	0.0152	0.170	0.016	10.625
16	5.18	4.95	96%	0.0148	0.172	0.014	12.286
16	5.18	4.72	91%	0.0142	0.170	0.012	14.167
16	5.18	5.08	98%	0.0153	0.170	0.010	17.000
17	2.59	2.53	98%	0.0076	0.090	0.017	5.294
17	2.59	2.26	87%	0.0068	0.080	0.015	5.333
17	2.59	1.93	74%	0.0058	0.080	0.015	5.333
17	2.59	2.59	100%	0.0078	0.070	0.013	5.385
17	2.59	2.36	91%	0.0071	0.080	0.014	5.714
17	2.59	2.59	100%	0.0078	0.080	0.014	5.714
17	2.59	2.59	100%	0.0078	0.090	0.014	6.429
17	2.59	2.89	112%	0.0087	0.090	0.011	8.182
17	2.59	2.49	96%	0.0075	0.090	0.011	8.182
17	2.59	2.13	82%	0.0064	0.080	0.014	5.714
17	2.59	3.69	142%	0.0111	0.130	0.016	8.125

## Data Availability

Data will be made available on reasonable request to swc@microsampling-laboratory.co.uk.
